# High Thermoelectric Performance in Ti_3_C_2_T*_x_* MXene/Sb_2_Te_3_ Composite Film for Highly Flexible Thermoelectric Devices

**DOI:** 10.1002/gch2.202300032

**Published:** 2023-07-05

**Authors:** Yunhe Xu, Bo Wu, Chengyi Hou, Yaogang Li, Hongzhi Wang, Qinghong Zhang

**Affiliations:** ^1^ State Key Laboratory for Modification of Chemical Fibers and Polymer Materials College of Materials Science and Engineering Donghua University Shanghai 201620 China; ^2^ Engineering Research Center of Advanced Glasses Manufacturing Technology Ministry of Education College of Materials Science and Engineering Donghua University Shanghai 201620 China

**Keywords:** composite film, flexible thermoelectric, Sb_2_Te_3_, thermoelectric devices, Ti_3_C_2_T*_x_* MXene

## Abstract

Flexible thin‐film thermoelectric devices (TEDs) can generate electricity from the heat emitted by the human body, which holds great promise for use in energy supply and biomonitoring technologies. The p‐type Sb_2_Te_3_ hexagon nanosheets are prepared by the hydrothermal synthesis method and compounded with Ti_3_C_2_T*_x_* to make composite films, and the results show that the Ti_3_C_2_T*_x_* content has a significant impact on the thermoelectric properties of the composite films. When the Ti_3_C_2_T*_x_* content is 2 wt%, the power factor of the composite film reaches ≈59 µW m^−1^ K^−2^. Due to the outstanding electrical conductivity, high specific surface area, and excellent flexibility of Ti_3_C_2_T*_x_*, the composite films also exhibit excellent thermoelectric and mechanical properties. Moreover, the small addition of Ti_3_C_2_T*_x_* has a negligible effect on the phase composition of Sb_2_Te_3_ films. The TED consists of seven legs with an output voltage of 45 mV at Δ*T* = 30 K. The potential of highly flexible thin film TEDs for wearable energy collecting and sensing is great.

## Graphical abstract

A flexible thermoelectric device based on composite films of p‐type Ti_3_C_2_T*_x_*/Sb_2_Te_3_ is fabricated. The Ti_3_C_2_T*_x_*/Sb_2_Te_3_ composite films are not only freestanding, highly flexible, and mechanically stable, but their prepared flexible thermoelectric device with seven legs can output an open‐circuit voltage of 45 mV at a temperature difference of about 30 K.

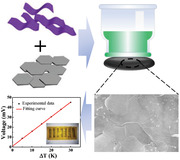

## 1 Introduction

Approximately 100–525 W of heat are released by the human body every day, making it a constant heat source.^[^ ^1^, ^2^, ^3^ ^]^ Therefore, reusing the heat emitted from the human body into electricity is beneficial to both energy conservation and environmental protection. One of the most promising solutions to the growing energy crisis and pollution‐related problems in modern society is thermoelectricity, an efficient, reliable, and clean energy conversion technology.^[^ ^4^, ^5^ ^]^

It is possible to evaluate a material's thermoelectric (TE) properties based on its dimensionless figure of merit factor *zT* = *S* ^2^ *σTκ* ^−1^, where *σ* and *S* represent the electrical conductivity and Seebeck coefficient, respectively.^[^ ^6^, ^7^, ^8^, ^9^ ^]^ Power factor (PF), an important indicator of thermoelectric performance, is often used to evaluate flexible thermoelectric materials.^[^ ^10^, ^11^, ^12^ ^]^ Some of the materials are with outstanding thermoelectric properties near room temperature such as Sb_2_Te_3_‐, Bi_2_Te_3_‐based alloys.^[^ ^13^, ^14^, ^15^, ^16^ ^]^ However, the rigidity and fragility of both block and thin‐film forms may lead to the failure of thermoelectric devices (TEDs) during long‐term use, limiting their application in wearable fields.^[^ ^17^ ^]^ Based on the experimental results and first principle study, the effect of grain size and thickness of Sb_2_Te_3_ on thermoelectric properties, higher thermoelectric properties can be obtained by introducing low‐dimensional structures through hydrothermal synthesis.^[^ ^18^, ^19^, ^20^, ^21^ ^]^ Nanoscale second phases, on the other hand, can provide rich heterogeneous interfaces and grain boundaries, which may profoundly affect the behavior of carriers and phonons in composites.^[^ ^22^ ^]^

In this sense, 2D nanomaterials, such as MXene, should show considerable effectiveness in improving TE properties because of their outstanding electrical conductivity, high specific surface area, and excellent flexibility. Other inorganic materials' thermoelectric properties can be modulated by MXene, such as Ti_3_C_2_T*_x_*.^[^ ^23^, ^24^, ^25^, ^26^ ^]^ Lu et al. compounded MXene with (Bi, Sb)_2_Te_3_, exhibiting excellent thermoelectric properties (*zT* of 1.23). After the addition of MXene, the Seebeck coefficient was basically unchanged, while the electrical conductivity was significantly increased.^[^ ^23^ ^]^ Exhibiting hydrophilic and metallic transport behavior, MXene has a remarkable electrical conductivity of 4600 S cm^−1^, making it an ideal material for supercapacitors, solar cells, and lithium‐ion batteries.^[^ ^27^ ^]^ However, MXene is not well suited for manufacturing composites, especially in combination with 2D materials. As a result of their ultrahigh surface area, 2D materials tend to aggregate. Moreover, it is not uncommon for 2D materials to decompose when subjected to high mechanical energy or high temperatures. Therefore, it is imperative to find a simple and effective composite method to prepare flexible thermoelectric materials.

In this study, we chose MXene as an effective component to improving the thermoelectric properties of Sb_2_Te_3_‐based alloys. We created p‐type flexible Ti_3_C_2_T*_x_*/Sb_2_Te_3_ composite films on polyimide (PI) substrates using an abstraction followed by a hot pressing process, and Sb_2_Te_3_ nanosheets of uniform size using a hydrothermal synthesis method. Using Sb_2_Te_3_ as the matrix, we fabricated Ti_3_C_2_T*_x_*/Sb_2_Te_3_ composites by adding the highly conductive MXene of Ti_3_C_2_T*_x_* second phase. Sb_2_Te_3_ as a 2D topological insulator due to its high Seebeck coefficient, at room temperature with excellent thermoelectric properties, and the Ti_3_C_2_T*_x_* MXene network as a conductive backbone that improves the electrical conductivity, thermoelectric properties, and mechanical properties of the composite films. The PF of the composite films reached 59 µW m^−1^ K^−2^ when the Ti_3_C_2_T*_x_* content was 2 wt%. In addition, flexible thermoelectric films can be used for the fabrication of TEDs. The TED consists of seven legs with an output voltage of 45 mV at Δ*T* = 30 K.

## 2 Results and Discussions

### 2.1 Material Synthesis and Device Fabrication

**Figure 1:**
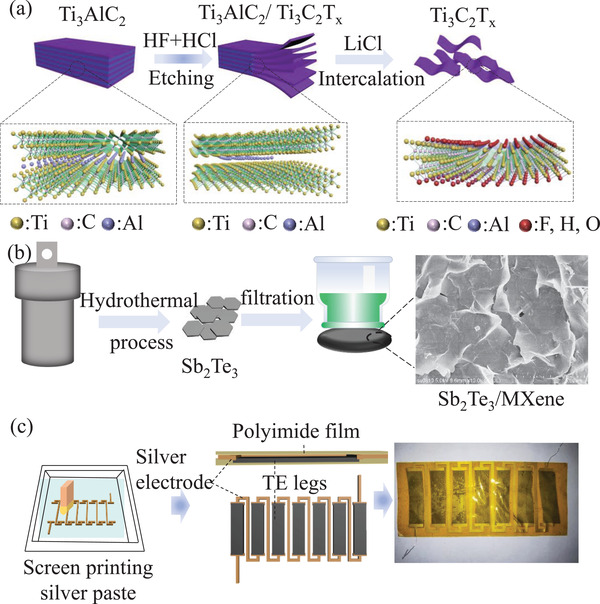
a) Schematic illustration of Ti_3_C_2_T*_x_* nanosheet synthesis process. b) Schematic diagram of Sb_2_Te_3_ nanosheet preparation and Ti_3_C_2_T*_x_*/Sb_2_Te_3_ composite film preparation process. c) TED manufacturing and structure schematic.

**Figure** **1**a shows a schematic diagram of the Ti_3_C_2_T*_x_* MXene synthesis process. The ternary carbide precursor powder (Ti_3_AlC_2_) was first chemically etched by a mixture of acids (HF + HCl) to break the metal M—A bond.^[^ ^28^ ^]^ After washing several times in deionized water, this multilayered MXene (HF‐etched powder) is further reacted with LiCl to insert lithium cations (Li^+^) between the negatively charged MXene sheets, resulting in the dislodging of the 2D MXene suspension, followed by further washing and sonication. As shown in Figure 1b, we used a simple method to prepare Sb_2_Te_3_ nanosheets with uniform dimensions by hydrothermal synthesis and p‐type flexible Ti_3_C_2_T*_x_*/Sb_2_Te_3_ composite films on filter membranes using a low‐temperature hot pressing process after extraction. The detailed preparation procedure is explained in the Experimental Section. As shown the Figure 1c, we prepared flexible TEDs with parallel structures using Ti_3_C_2_T*_x_*/Sb_2_Te_3_ composite films. Due to the potential toxicity of thermoelectric nanomaterials, a PI film was used to encapsulate the entire thermoelectric active layer. The PI film separates the human skin from the thermoelectric material when worn by the human body. For future large‐scale production, a simple fabrication process was chosen for TED fabrication. First, the conductive silver paste is passed through a custom screen printing stencil and the electrode patterns are all printed on the PI film. After curing, seven Ti_3_C_2_T*_x_*/Sb_2_Te_3_ films cut to 8 mm wide and 30 mm long are coated with silver paste at both ends, connecting the cut thermoelectric legs in series along the screen‐printed electrode pattern.

### 2.2 Phase and Microstructures

**Figure 2:**
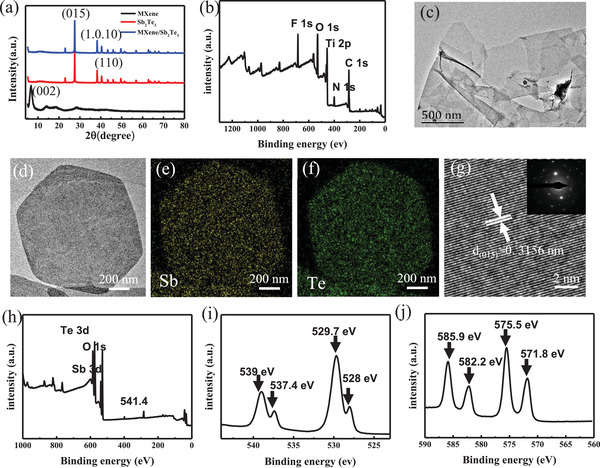
a) XRD patterns from pure Sb_2_Te_3_ nanosheet, a Ti_3_C_2_T*_x_*/Sb_2_Te_3_ composite film, and Ti_3_C_2_T*_x_* film. b) XPS spectrum of Ti_3_C_2_T*_x_* nanosheet. c) TEM images of Ti_3_C_2_T*_x_* nanosheet. d) TEM images of Sb_2_Te_3_ nanosheet. e,f) Images of the SAED mapping of the Sb_2_Te_3_ nanosheet. g) High‐resolution TEM picture of Sb_2_Te_3_ nanosheet. h) XPS spectrum of Sb_2_Te_3_ nanosheet. i) High‐resolution XPS spectrum of the Sb peak and its oxidized state scan of the Sb_2_Te_3_ nanosheet. j) High‐resolution XPS spectrum of the Te peak and its oxidized state scan of the Sb_2_Te_3_ nanosheet.

Ti_3_C_2_T*_x_* MXene films were prepared by vacuum filtration and their phase composition was analyzed by X‐ray diffraction (XRD). The two characteristic peaks of Ti_3_C_2_T*_x_* MXene can be clearly identified as the (002) lattice plane at 6.6° and the (004) lattice plane at 13.6°, respectively. The XRD pattern of Ti_3_C_2_T*_x_*/Sb_2_Te_3_ composite (**Figure** **2**a) is consistent with the Sb_2_Te_3_ phase (JCPDS no.15‐0874). No peaks belonging to Ti_3_C_2_T*_x_* were observed in the XRD patterns of the mixed films, which may be due to the low content of Ti_3_C_2_T*_x_*.

The XRD results of the Sb_2_Te_3_ and 2 wt% Ti_3_C_2_T*_x_*/Sb_2_Te_3_ composites showed almost identical lattice parameters, which also demonstrated that the small addition of Ti_3_C_2_T*_x_* has a negligible impact on the phase constitution of the Sb_2_Te_3_ films. The elemental composition of Ti_3_C_2_T*_x_* was further investigated using X‐ray photoelectron spectroscopy (XPS) (Figure 2b). As seen in the XPS pattern, Ti, C, O, and F atoms were observed in the etched Ti_3_C_2_T*_x_* MXene, and no Al elements were detected, proving that the acid completely etched the Al elements in the MAX (Ti_3_AlC_2_) phase.^[^ ^29^ ^]^ As shown in high‐resolution transmission electron microscope (HRTEM) image, there are many folds around the edges of Ti_3_C_2_T*_x_*, which adequately indicates that prepared Ti_3_C_2_T*_x_* is soft and ultrathin (Figure 2d). In view of the excellent application of nanostructuring strategies in improving TE performance, Sb_2_Te_3_ nanosheets were successfully synthesized in bulk by a modified hydrothermal method. The EDS mapping of typical nanosheets showed uniform distributions of Sb and Te elements (Figure 2e,f). From the HRTEM photograph (Figure 2g), it is observed that the crystalline surface spacing of the sample is about 0.3156 nm, which matches the hexagonal Sb_2_Te_3_(015) crystalline surface spacing. The corresponding selected area electron diffraction (SAED) (Figure 2g inset) indicates that the Sb_2_Te_3_ nanosheets have a single‐crystal structure. The synthesized Sb_2_Te_3_ nanosheets were further investigated by XPS. Figure 2h shows the XPS measured spectra of the prepared Sb_2_Te_3_ nanosheets, and the peaks correspond to show various signals of Sb_2_Te_3_ nanosheets. Shown in Figure 2i,j are the XPS patterns of the Sb peak of Sb_2_Te_3_ nanosheets, the Te peak, and their oxidation states. The peaks of Sb‐3d_5/2_ and Sb‐3d_3/2_ appear at 528 and 537.4 eV, respectively. The peak of Te‐3d_5/2_ at 571.8 eV and the peak of Te‐3d_3/2_ at 582.2 eV are clearly seen in the Figure 2j, which is in accordance with the literature XPS results reported for Sb_2_Te_3_.^[^ ^30^ ^]^ In addition, a second set of peaks can be seen at higher binding energies of 539 and 529.7 eV for Sb‐3d_5/2_ and Sb‐3d_3/2_, which are oxidation peaks formed due to surface oxidation. For tellurium, there is also a second set of weak peaks at higher binding energies, attributed to surface oxidation peaks of Te‐3d_3/2_ (585.9 eV) and Te‐3d_5/2_ (575.5 eV), respectively.

### 2.3 Phase Interface Characteristics

**Figure 3:**
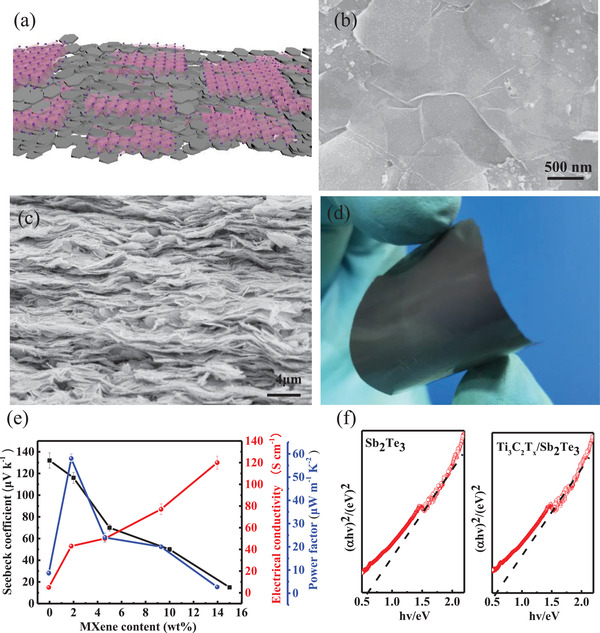
a) A Schematic diagram showing the stacking of Ti_3_C_2_T*_x_* and Sb_2_Te_3_ in the Ti_3_C_2_T*_x_*/Sb_2_Te_3_ composite films. b) Surface SEM images of the Ti_3_C_2_T*_x_*/Sb_2_Te_3_ composite film. c) Cross‐sectional SEM images of the Ti_3_C_2_T*_x_*/Sb_2_Te_3_ composite film. d) Digital picture of the Ti_3_C_2_T*_x_*/Sb_2_Te_3_ composite film. e) Plots of Seebeck coefficient, conductivity, and PF increasing with increasing Ti_3_C_2_T*_x_* content (wt%). f) The optical bandgaps of the Sb_2_Te_3_ and Ti_3_C_2_T*_x_*/Sb_2_Te_3_ composite films.

According to the literature, Ti_3_C_2_T*_x_* has ultrahigh conductivity, and its 2D structure makes it flexible.^[^ ^31^ ^]^ The addition of Ti_3_C_2_T*_x_* to Sb_2_Te_3_ not only enhances the flexibility of the composite film but also enhances its conductivity by providing an efficient transmission route for the carrier. As shown in **Figure** **3**a–c, Ti_3_C_2_T*_x_* is in intimate touch with the interfaces of Sb_2_Te_3_ nanosheets, and this contact greatly improves the mechanical properties of the composite films, allowing them to bend without shattering. As can be seen from the digital photographs in Figure 3d, the bent‐independent Ti_3_C_2_T*_x_*/Sb_2_Te_3_ composite film has a metallic luster and is held bent in the hand without shattering. It can also be seen in Figure 3a–c that the composite film is formed by stacking many 2D lamellae, forming plenty of contact surfaces. It follows that the interface between Ti_3_C_2_T*_x_* and Sb_2_Te_3_ in the composite film can be considered a typical metal–semiconductor contact, and this contact interface is the main cause of the thermal and electrical transport variations. Therefore, we suggest that the carriers and phonons in Ti_3_C_2_T*_x_*/Sb_2_Te_3_ films have a special transport mechanism. The presence of Ti_3_C_2_T*_x_* MXene facilitates the formation of conductive network channels for carrier transport, while the physical contact between Ti_3_C_2_T*_x_* and Sb_2_Te_3_ improves phonon scattering and makes phonon scattering simpler and more convenient. In addition, field emission scanning electron microscope (FESEM) images (Figure 3c) show Ti_3_C_2_T*_x_* MXene embedded in Sb_2_Te_3_, which indicates the formation of an effective carrier channel.

### 2.4 Performance of Ti_3_C_2_T*_x_*/Sb_2_Te_3_ Composite Film

The variation curves of the thermoelectric properties of the composite film samples with Ti_3_C_2_T*_x_* and MXene content are shown in Figure 3e,f. From the PF equation, it can be seen that conductivity, Seebeck coefficient are the key factors determining the thermoelectric properties of the material. Shown in Figure 3e is the variation of conductivity and Seebeck coefficient of Ti_3_C_2_T*_x_*/Sb_2_Te_3_ composite films with Ti_3_C_2_T*_x_* MXene content. The Seebeck coefficient of the p‐type Sb_2_Te_3_ thermoelectric film without Ti_3_C_2_T*_x_* MXene addition is about 131 µV K^−1^. When the Ti_3_C_2_T*_x_* MXene addition exceeded 2 wt%, the Seebeck coefficient values decreased with increasing Ti_3_C_2_T*_x_* MXene content. The Seebeck coefficient of the composite film has decreased to 18 µV K^−1^ when the Ti_3_C_2_T*_x_* addition is increased to 8 wt%. The red curve of Figure 3e shows the gradual increment in electrical conductivity of the composite film with the increased Ti_3_C_2_T*_x_* MXene content. As the Ti_3_C_2_T*_x_* MXene content increases from 0 to 8 wt%, the conductivity increases from 10 to 120 S cm^−1^ due to the very high conductivity of Ti_3_C_2_T*_x_* MXene (4600 S cm^−1^). For metals or semiconductors, the conductivity equation is as following Equation (1)

(1) $$\begin{array}{c} \sigma \;=\;ne\mu \end{array}$$

where *σ*, *n*, *e*, and *µ* are the conductivity, carrier density, charge per carrier (elementary charge), and carrier mobility, respectively. It can be seen from the equation that the conductivity increases with increasing carrier concentration. Therefore, as the Ti_3_C_2_T*_x_* MXene content increases, the carrier concentration in the composite film increases, the mobility rises, and the conductivity increases. However, the Seebeck coefficient of the composite film is opposite to the conductivity, which decreases as the carrier concentration increases. The relationship between Seebeck coefficient and carrier concentration can be seen from a relatively simple electron transfer model, which is as following Equation (2)

(2) $$\begin{array}{c} S\;=\;\frac{8\pi^{2}K_{B}^{2}}{3eh^{2}}m^{*}T{\left(\frac{\pi }{3n}\right)}^{\frac{2}{3}} \end{array}$$

where *k*_B_ and *h* are the Boltzmann constant and Planck constant, and *m^∗^* = 0.58 *m*_e_. This relationship has been widely used in composites.^[^ ^14^ ^]^ With the increase in Ti_3_C_2_T*_x_* MXene content, the carrier concentration increases significantly. It can be seen from the figure that the Seebeck coefficient of the composite film reduces with the addition of Ti_3_C_2_T*_x_* MXene content, which may be mainly due to the obvious increase in carrier concentration. Thus, the introduction of Ti_3_C_2_T*_x_* MXene changes the energy bandgap range of Sb_2_Te_3_, and the carrier concentration increases with the increase of Ti_3_C_2_T*_x_* content, with a consequent decrease of the Seebeck coefficient.^[^ ^26^ ^]^ 2D Sb_2_Te_3_ nanosheets and Ti_3_C_2_T*_x_* MXene have different work functions, which create potential barriers at the interface between Sb_2_Te_3_ nanosheets and Ti_3_C_2_T*_x_* MXene and disperse the lower‐energy carriers, so that the decrease in Seebeck coefficient is not significant when a small amount of Ti_3_C_2_T*_x_* is added to Sb_2_Te_3_.^[^ ^23^ ^]^ The results show that the insignificant decrease in Seebeck coefficient for small amounts of composite Ti_3_C_2_T*_x_* film is mainly on account of the paradoxical decrease in carrier concentration and the filtering effect of low‐energy carriers. As shown in Figure 3e, the introduction of Ti_3_C_2_T*_x_* MXene as the second phase enhanced the conductivity of the composite film with its ultrahigh conductivity, while the Seebeck coefficient was moderately reduced, therefore the PF showed a trend of first increasing and then decreasing. The PF reaches 59 µW m^−1^ K^−2^ when Ti_3_C_2_T*_x_* is 2 wt%, which is 6 times higher than that of the pure Sb_2_Te_3_ film. Here, we compared the power factors in this study with those shown in the literature (**Table** **1**). In the table, Eguchi et al.^[^ ^32^ ^]^ prepared hybrid films of SWCNT and Sb_2_Te_3_ nanosheets by electrodeposition and their power factor reached 59.5 µW m^−1^ K^−2^. In the present study, although electrodeposition was not performed, the maximum power factor reaching 59 µW m^−1^ K^−2^ is comparable to the maximum value in the literature. At 300 K, the *zT* value of the MXene/Sb_2_Te_3_ composite membrane was 1.8 × 10^−3^, which was better than that of the pure Sb_2_Te_3_ membrane (1.1 × 10^−3^).

**Table 1:** Room‐temperature TE properties of some reported flexible TE films and our films

Material	*S* [µV K^−1^]	*σ* [S cm^−1^]	PF [µW m^−1^ K^−2^]	Ref.
Ti_3_C_2_T*_x_*/Sb_2_Te_3_	116	43.8	59	This work
SWCNTs/Sb_2_Te_3_	60	154	55	[22]
SWCNTs/Sb_2_Te_3_	40	322	59.5	[32]
Bi_2_Te_3_/PEDOT:PSS	15.1	423	9.9	[33]
Bi_2_Te_3_/CCF	15	5	0.15	[34]
Bi_2_Te_3_/PEDOT:PSS	≈16	1295.21	32.26	[35]
Bi_0.5_Sb_1.5_Te_3_/PLA	199	1.73	6.8	[36]
Bi_0.5_Sb_1.5_Te_3_/MWCNTs/PLA	178.7	3.54	11.3	[36]
Cu_1.75_Te/PVDF	9.6	2490	23	[37]

Note: Single‐walled carbon nanotubes (SWCNTs), Poly(3, 4‐ethylenedioxythiophene)/poly(styrenesulfonate)(PEDOT:PSS), Polylactic acid (PLA), Multi‐walled carbon nanotubes (MWCNTs), Polyvinylidene difluoride (PVDF), MWCNT disperions were first drop‐coated on the cotton fabric substrate (CCF).

According to the theory of Tauc plot,^[^ ^38^ ^]^ the optical bandgap can be estimated by the following Equation (3)

(3) $$\begin{array}{c} \left(\alpha ua\right)=A{\left(h\nu -E_{g}\right)}^{n} \end{array}$$

where *hν* is the photon energy and *A* is a constant determined by the effective mass. The exponent *n* depends on the electron‐jumping properties of the material. Depending on its type, the value of *n* is a constant 1/2 for Sb_2_Te_3_ films that directly allow a narrow bandgap.^[^ ^39^ ^]^ As shown in Figure 3f, the optical bandgap decreases from 0.603 to 0.589 eV after the addition of MXene 2 wt%.

### 2.5 Device Performance

**Figure 4:**
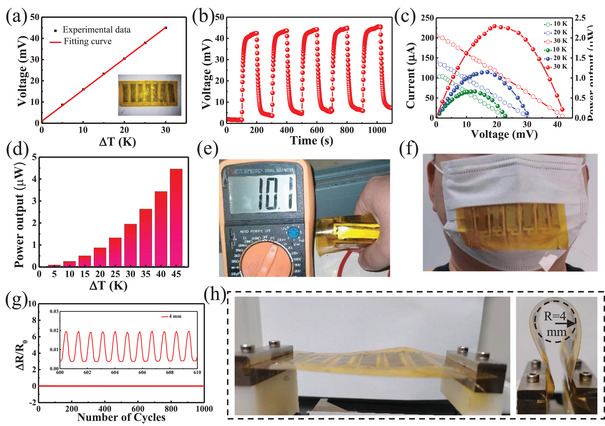
a) Open voltage curve at different temperature differences. b) Open voltage cycle test under ∆*T* = 30 K c) TED current and power output versus voltage curve at different external resistances, at ∆*T* = 10, 20, and 30 K, respectively. d) Histogram of TED output power at different temperature ranges. e) Photos of the TED palm temperature generating power. f) Photos of the TED breathing temperature power generation. g) Normalized resistance changes (Δ*R*/*R*_0_) in the measurements were repeatedly bent 1000 times with a thermoelectric device at a bend radius of 4 mm. The illustration shows an enlarged view of some of the data. h) Photos showing the bending of the hybrid films.

**Figure** **4**a shows the open voltage of the TED under different temperature differences. It can be clearly seen from the figure that with the increasing temperature difference, the open voltage increases, and the red line in the figure is the fitted curve, the open voltage increases linearly with the temperature difference. The open voltage reaches 45 mV at Δ*T* = 30 K. As shown in Figure 4b, it is the curve of the output voltage of the TED with time when Δ*T* = 30 K is applied alternately. From the figure, it can be seen that when the temperature difference generating voltage is applied to the TED, the removal of the temperature difference voltage disappears, and the open voltage is stable at about 45 mV at a 30 K temperature difference with good cycling performance. Figure 4c shows the current and power versus voltage curves of the TED at different temperature differences (Δ*T* = 10, 20, and 30 K). At Δ*T* = 30 K, the output voltage of the TED with 7 thermoelectric legs is 21.4 mV, the output current is 0.106 mA, and the output power is 2.26µW. From the Equation (4)

(4) $$\begin{array}{c} P\;=\;\frac{E^{2}}{\frac{{\left(R\;-\;r\right)}^{2}}{R}\;+\;4r}\; \end{array}$$

From Equation (3), it can be seen that to maximize the output power of the TED, the *r* should be the same as the *R* of the TED. According to Figure 4c, the load resistance ranges between 201 and 256 Ω. The calculated results are basically the same as the measured value of the *R* of the TED. The measurement of the maximized output power of the TED at different temperature ranges is done by selecting a fixed resistance. The output power is 4.46 µW when the TED temperature gradient is 45 K using 200 Ω as an external resistor (Figure 4d). From this, it can be further inferred that if the power‐using device requires higher power, the TED can be obtained by increasing the temperature difference.

As shown in Figure 4e, the TED is held curled up in the hand, and the temperature difference between the palm temperature and the environmental temperature produces an open‐circuit voltage of 10.1 mV. The TED is applied to a mask (Figure 4f), which is worn as a thermal barrier to impede heat transfer, ensuring a temperature difference along the plane of the TED, thus producing a temperature difference at both ends of the TED that varies regularly and continuously with the heat generated by breathing, the voltage also varies regularly (Video S1, Supporting Information). The video shows that the TED has a variety of uses, not only for heat acquisition but also as a sensor for body health detection.

We have also experimentally demonstrated the mechanical reliability of the resistance change of the thermoelectric device under bending conditions with a 4 mm radius of curvature. We demonstrated that the TED can maintain stable electrical properties after repeated mechanical bending, where the normalized resistance varies for 1000 cycles at a 4 mm radius of curvature (Figure 4g,h). It is noteworthy that the internal resistance variation of the TED remains below 2%, which implies a stable mechanical reliability of the assembled thermoelectric devices.

## 3 Conclusion

In summary, we prepared Ti_3_C_2_T*_x_*/Sb_2_Te_3_ composite films and successfully fabricated TEDs for thermoelectric applications. The good thermoelectric properties of the composite films (PF ≈ 59 µW m^−1^ K^−2^) were ascribed to the excellent conductivity of Ti_3_C_2_T*_x_* and excellent thermoelectric properties of Sb_2_Te_3_ nanosheets. We fabricated a TED consisting of seven legs with an open voltage of ≈45 mV at Δ*T* = 30 K. This work shows that Ti_3_C_2_T*_x_* MXene material, as an effective second phase for nanocomposite with thermoelectric materials, has great potential for tuning the electrical and thermal properties of thermoelectric materials to achieve extremely high energy conversion efficiency. Moreover, the small addition of Ti_3_C_2_T*_x_* has a negligible effect on the phase composition of Sb_2_Te_3_ films. These results indicate that MXene composites with thermoelectric materials have great promise for application in wearable TEDs.

## 4 Experimental Section

### Materials

High purity (analytical reagent) SbCl_3_ (99%), TeO_2_ (99.99%), LiCl (99.99%), and Polyvinylpyrrolidone (PVP) were supplied by the Titan Scientific Co. Ltd. Ethylene glycol (≥99%), NaOH (96%), N_2_H_4_·H_2_O (16 m),HF (14 m), and HCl (9 m) were supplied by Sinopharm Chemical Reagent Co., Ltd. The Ti_3_AlC_2_ powders were supplied by Jilin 11 technology Co., Ltd.

### Preparation of Ti_3_C_2_T*_x_* MXene Nanosheet

To prepare monolayer Ti_3_C_2_T*_x_* MXene nanosheets, the MAX phase (Ti_3_AlC_2_) was first etched by acid etching, followed by intercalation, and finally combined and sonicated for exfoliation. Specifically, H_2_O (6 mL), hydrochloric acid (12 mL, 9 m), and HF (2 mL, 14 m) were mixed well. Then, Ti_3_C_2_T*_x_* powder (1.0 g) was slowly incorporated into the acid mixture solution, which was evenly and continuously stirred. After a 24 h reaction, the etched solution was cleaned with deionized water (DI), at 3500 rpm for 5 min, and the process was repeated 3–4 times. The centrifuged precipitate was then transferred to deionized water (70 mL) with LiCl (1 g), shaken for 1 h using a shaker, and washed again with DI. To exfoliate the obtained multilayers of Ti_3_C_2_T*_x_* into monolayers, the precipitate was transferred to DI (100 mL) and treated with ultrasound under argon bubbling conditions for 2 h. Finally, it was centrifuged at 3500 rpm for 30 min, discarding the black precipitate at the bottom of the centrifuge tube, and the dark green solution was collected as the dispersion of monolayer Ti_3_C_2_T*_x_* MXene nanosheets (10 mg mL^−1^). It was placed in a 4 °C refrigerator to store.

### Synthesis of Sb_2_Te_3_ Nanosheet

First, 140 mL of ethylene glycol was poured into a 200 mL round bottom flask, and then TeO_2_ (2.8728 g), SbCl_3_ (2.7900 g), and PVP (1.6 g) were added. Then, the pH of the miscible liquids was adjusted by adding NaOH (2.92 g). The miscible liquids was kept at 35 °C and stirred for 5 h until a clear liquid was formed. After the solution was clear, N_2_H_4_·H_2_O (16 mL) was added and stirred magnetically for 10 min to obtain a brown, clear liquid. The mixture was put into three Teflon‐lined stainless steel reactors (100 mL) and held in an oven at 180 °C for 10 h. When the miscible liquids were cooled to room temperature, the reaction solution was washed by centrifuge; DI, acetone, and anhydrous ethanol were used sequentially several times, and then they were dried in a 60 °C vacuum oven for 12 h.

### Fabrication of TED

The Ti_3_C_2_T*_x_*/Sb_2_Te_3_ composite films were prepared in three simple steps. Step 1 was solvent mixing, where Ti_3_C_2_T*_x_* and Sb_2_Te_3_ were uniformly mixed by an ultrasonic machine; step 2 was vacuum filtration, where the uniformly mixed solution was evacuated; step 3 was hot pressing, where the evacuated composite films were hot‐pressed by a hot press (120 °C, 5 MPa, 3–5 min). The hot‐pressed mixed film was then cut into seven uniformly long (30 × 8 mm^2^) to make TED. Single‐sided PI tape was used as the substrate. The TEDs were first fabricated by screen printing a highly conductive silver paste on a single‐sided adhesive PI tape. After the silver paste had dried and set, the thermoelectric legs were strung together and finally encapsulated with another single‐sided adhesive polyimide tape.

### Characterization and Measurements

The surface and cross‐sectional topography of films were measured on a scanning electron microscope (SEM). The surface morphology and elemental distribution of Sb_2_Te_3_ nanosheets and Ti_3_C_2_T*_x_* were observed using a transmission electron microscope (TEM). XPS was used to analyze the binding energy within Sb_2_Te_3_ nanosheets, Ti_3_C_2_T*_x_*, and composite films. XRD was used to examine the structures of Sb_2_Te_3_ nanosheets, Ti_3_C_2_T*_x_*, and composite films at 300 mA and 40 kV with Cu K irradiation (= 1.5406). Open‐circuit voltages, load voltages, currents, and temperatures were captured using a SourceMeter (Keithley 2700). The conductivity of the samples was measured with a four‐point probe system. The Seebeck coefficient was determined using a handheld Seebeck meter (PTM‐3, Wuhan Jiayitong Technology Co., Ltd.).

## Conflict of Interest

The authors declare no conflict of interest.

## Supporting information

Supplemental Video 1

## Data Availability

The data that support the findings of this study are available from the corresponding author upon reasonable request.
